# Supplementary material for: The association between use of proton-pump inhibitors and excess mortality after kidney transplantation: A cohort study
Source: PLoS Med. 2020 Jun 15;17(6):e1003140. doi: 10.1371/journal.pmed.1003140 (PMC7295199; doi:10.1371/journal.pmed.1003140)
Supplement: S1 Appendix — (DOCX) [file pmed.1003140.s003.docx]

**II VRAGEN OVER UW LEEFSTIJL**

**1 Roken**

Rookt u nu, of hebt u ooit gerookt? (zo nee, ga naar vraag…) □ Nee □ Ja

Hoeveel rookt u momenteel gemiddeld

Aantal sigaretten per dag _____ of aantal sigaretten per week _________

Aantal shagjes per dag _____ of aantal shagjes per week _________

Aantal cigarillo’s per dag _____ of aantal cigarillo’s per week _________

Aantal sigaren per dag _____ of aantal sigaren per week _________

Pijp tabak in gram/dag _____ of pijptabak in gram/week _________

Hebt u ooit gedurende een volledig jaar gerookt? □ Nee □ Ja

Hoe oud was u toen u begon te met roken? _____ Jaar

Bent u gestopt met roken? Zo nee, ga naar vraag …) □ Nee □ Ja

Hoe oud was u toen u stopte met roken? _____ Jaar

Hoeveel hebt u gerookt tot nu toe?

(bv. van uw 16^e^ tot uw 20^e^ rookte u 10 sigaretten/~~cigarillo’s~~/~~sigaren~~/~~gram pijptabak~~* per dag

Van uw 20^e^ tot uw 30^e^ rookte u 5 ~~sigaretten~~/~~cigarillo’s~~/sigaren/~~gram pijptabak~~* per dag, etc)

Van uw ….^e^ tot uw …..^e^ rookte u …… sigaretten/shagjes/ cigarillo’s/sigaren/gram pijptabak* per dag

Van uw ….^e^ tot uw …..^e^ rookte u …… sigaretten/shagjes/ cigarillo’s/sigaren/gram pijptabak* per dag

Van uw ….^e^ tot uw …..^e^ rookte u …… sigaretten/shagjes/ cigarillo’s/sigaren/gram pijptabak* per dag

Van uw ….^e^ tot uw …..^e^ rookte u …… sigaretten/shagjes/ cigarillo’s/sigaren/gram pijptabak* per dag

Van uw ….^e^ tot uw …..^e^ rookte u …… sigaretten/shagjes/ cigarillo’s/sigaren/gram pijptabak* per dag

* Doorhalen wat niet van toepassing is

Inhaleert u of inhaleerde u tijdens het roken? □ Nee □ Ja

**Passief roken als volwassene**

Bent u in de afgelopen 12 maanden regelmatig blootgesteld geweest aan tabaksrook □ Nee □ Ja

van anderen

Hoeveel mensen roken regelmatig in uw huishouden, uzelf niet meegerekend? _____ mensen

Rookt men regelmatig in de ruimte waar u werkt? □ Niet van toepassing want ik heb geen werk

□ Nee

□ Ja

Hoeveel uur per dag wordt u blootgesteld aan de tabaksrook van anderen? _____ uur

**2 Alcohol**

63 a) Hoe vaak heeft u de afgelopen maand **bier** gedronken? Denk bijvoorbeeld aan pils, witbier, bokbier, dubbel/tripel.

0 Deze maand niet → ga naar vraag 64

0 1 dag per maand

0 2-3 dagen per maand

0 1 dag per week

0 2 dagen per week

0 3 dagen per week

0 4 dagen per week

0 5 dagen per week

0 6 dagen per week

0 7 dagen per week

63 b) **Waarin** zat het bier?

|  | nooit | soms | vaak | altijd |
| --- | --- | --- | --- | --- |
| Flesje of blikje | 0 | 0 | 0 | 0 |
| Beugelfles of halve liter | 0 | 0 | 0 | 0 |
| Glas | 0 | 0 | 0 | 0 |

63 c) Hoeveel **glazen/flesjes/blikjes** dronk u gemiddeld op zo’n dag?

0 1

0 2

0 3

0 4

0 5

0 6

0 7

0 8

0 9

0 10

0 11

0 12

64 a) Hoe vaak dronk u de afgelopen maand **alcoholarm** of **alcoholvrij bier**?

0 Deze maand niet → ga naar vraag 65

0 1 dag per maand

0 2-3 dagen per maand

0 1 dag per week

0 2 dagen per week

0 3 dagen per week

0 4 dagen per week

0 5 dagen per week

0 6 dagen per week

0 7 dagen per week

64 b) Hoeveel **flesjes of blikjes** dronk u gemiddeld op zo’n dag?

0 1

0 2

0 3

0 4

0 5

0 6

0 7

0 8

0 9

0 10

0 11

0 12

65a) Hoe vaak heeft u de afgelopen maand **wijn, sherry, port of vermouth** gedronken?

0 Deze maand niet → ga naar vraag …

0 1 dag per maand

0 2-3 dagen per maand

0 1 dag per week

0 2 dagen per week

0 3 dagen per week

0 4 dagen per week

0 5 dagen per week

0 6 dagen per week

0 7 dagen per week

65 b) Hoeveel **glazen** dronk u gemiddeld op zo’n dag?

0 1

0 2

0 3

0 4

0 5

0 6

0 7

0 8

0 9

0 10

0 11

0 12

66 a) Hoe vaak heeft u de afgelopen maand **advocaat** genuttigd?

0 Deze maand niet → ga naar vraag 67

0 1 dag per maand

0 2-3 dagen per maand

0 1 dag per week

0 2 dagen per week

0 3 dagen per week

0 4 dagen per week

0 5 dagen per week

0 6 dagen per week

0 7 dagen per week

66 b) Hoeveel **glazen** nam u gemiddeld op zo’n dag?

0 1

0 2

0 3

0 4

0 5

0 6

0 7

0 8

0 9

0 10

0 11

0 12

67 a) Hoe vaak heeft u de afgelopen maand **mixdranken** gedronken? Denk bijvoorbeeld aan alcoholische drank gemengd met frisdrank of vruchtensap zoals breezer en rum-cola.

0 Deze maand niet → ga naar vraag 68

0 1 dag per maand

0 2-3 dagen per maand

0 1 dag per week

0 2 dagen per week

0 3 dagen per week

0 4 dagen per week

0 5 dagen per week

0 6 dagen per week

0 7 dagen per week

67 b) Hoeveel **glazen/ blikjes/ flesjes**  dronk u gemiddeld op zo’n dag?

0 1

0 2

0 3

0 4

0 5

0 6

0 7

0 8

0 9

0 10

0 11

0 12

68a) Hoe vaak dronk u de afgelopen maand **sterke drank**? Denk bijvoorbeeld aan jenever, whisky, rum, gin, cognac, vieux en likeur? Denk ook aan likeur in of bij de koffie.

0 Deze maand niet → ga naar vraag …

0 1 dag per maand

0 2-3 dagen per maand

0 1 dag per week

0 2 dagen per week

0 3 dagen per week

0 4 dagen per week

0 5 dagen per week

0 6 dagen per week

0 7 dagen per week

68 b) Hoeveel **glazen** dronk u gemiddeld op zo’n dag?

0 1

0 2

0 3

0 4

0 5

0 6

0 7

0 8

0 9

0 10

0 11

0 12
