# Supplementary material for: The association between use of proton-pump inhibitors and excess mortality after kidney transplantation: A cohort study
Source: PLoS Med. 2020 Jun 15;17(6):e1003140. doi: 10.1371/journal.pmed.1003140 (PMC7295199; doi:10.1371/journal.pmed.1003140)
Supplement: S1 Table — Results from analyses in the nonimputed dataset from the TransplantLines study. Model 1: PPI use adjusted for age, sex, BMI, time since transplantation. Model 2: Model 1 additionally adjusted for eGFR, proteinuria, deceased donor transplant, preemptive transplantation, primary renal disease. Model 3: Model 2 additionally adjusted for donor age, donor sex, donor weight, donor height, donor serum creatinine, number of HLA mismatches, and induction therapy. Model 4: Model 2 additionally adjusted for smoking behavior and alcohol use. Model 5: Model 2 additionally adjusted for use of antihypertensive agents, platelet inhibitors, vitamin K antagonists, proliferation inhibitors, and CNIs. Model 6: Model 2 additionally adjusted for comorbidities (diabetes, history of cardiovascular disease). Model 7: Model 2 additionally adjusted for potential mediators (plasma magnesium and serum iron). (DOCX) [file pmed.1003140.s004.docx]

**S1 Table**. Association of PPI use with mortality in 703 stable KTRs. Results from analyses in the non-imputed dataset from the TransplantLines study.

|  |  | All-Cause Mortality | | |
| --- | --- | --- | --- | --- |
| No. events = 194 |  | HR | 95% CI | *P* |
| Crude |  | 1.86 | 1.38 – 2.52 | <0.001 |
| Model 1 |  | 1.73 | 1.25 – 2.38 | 0.001 |
| Model 2 |  | 1.67 | 1.20 – 2.32 | 0.002 |
| Model 3 |  | 1.74 | 1.17 – 2.60 | 0.007 |
| Model 4 |  | 1.71 | 1.18 – 2.48 | 0.005 |
| Model 5 |  | 1.48 | 1.05 – 2.07 | 0.02 |
| Model 6 |  | 1.44 | 1.03 – 2.01 | 0.03 |
| Model 7 |  | 1.59 | 1.13 – 2.23 | 0.008 |

Model 1: PPI use adjusted for age, sex, BMI, time since transplantation. Model 2: Model 1 additionally adjusted for eGFR, proteinuria, deceased donor transplant, pre-emptive transplantation, primary renal disease. Model 3: Model 2 additionally adjusted for donor age, donor sex, donor weight, donor height, donor serum creatinine, number of HLA-mismatches and induction therapy.

Model 4: Model 2 additionally adjusted for smoking behavior and alcohol use. Model 5: Model 2 additionally adjusted for use of antihypertensive agents, platelet inhibitors, vitamin K antagonists, proliferation inhibitors and CNI. Model 6: Model 2 additionally adjusted for comorbidities (diabetes, history of cardiovascular disease). Model 7: Model 2 additionally adjusted for potential mediators (plasma magnesium and serum iron).
