# Supplementary material for: The association between use of proton-pump inhibitors and excess mortality after kidney transplantation: A cohort study
Source: PLoS Med. 2020 Jun 15;17(6):e1003140. doi: 10.1371/journal.pmed.1003140 (PMC7295199; doi:10.1371/journal.pmed.1003140)
Supplement: S2 Table — Model 1: PPI use adjusted for age, sex, time since transplantation. Model 2: Model 1 additionally adjusted for eGFR, deceased donor transplant, preemptive transplantation, primary renal disease. (DOCX) [file pmed.1003140.s005.docx]

**S2 Table**. Association of PPI use with cause-specific mortality in 703 stable KTRs.

| Cause of death |  | Cardiovascular diseases | | Infectious diseases | | Malignant diseases | | Miscellaneous causes | |
| --- | --- | --- | --- | --- | --- | --- | --- | --- | --- |
| Number of events |  | 72 | | 47 | | 36 | | 29 | |
|  |  | HR (95%CI) | *P* | HR (95%CI) | *P* | HR (95%CI) | *P* | HR (95%CI) | *P* |
| Crude |  | 2.42 (1.43 – 4.08) | <0.001 | 1.89 (1.02 – 3.49) | 0.04 | 1.53 (0.78 – 3.03) | 0.2 | 1.41 (0.74 – 2.69) | 0.3 |
| Model 1 |  | 2.18 (1.26 – 3.79) | 0.006 | 2.07 (1.07 – 4.00) | 0.03 | 1.56 (0.76 – 3.21) | 0.2 | 1.07 (0.54 – 2.12) | 0.9 |
| Model 2 |  | 2.01 (1.15 – 3.53 ) | 0.02 | 1.88 (0.96 – 3.71) | 0.07 | 1.65 (0.79 – 3.44) | 0.2 | 1.05 (0.50 – 2.19) | 0.9 |

Model 1: PPI use adjusted for age, sex, BMI, time since transplantation. Model 2: Model 1 additionally adjusted for eGFR, proteinuria, deceased donor transplant, pre-emptive transplantation, primary renal disease.
