# Supplementary material for: The association between use of proton-pump inhibitors and excess mortality after kidney transplantation: A cohort study
Source: PLoS Med. 2020 Jun 15;17(6):e1003140. doi: 10.1371/journal.pmed.1003140 (PMC7295199; doi:10.1371/journal.pmed.1003140)
Supplement: S3 Table — Model 1: PPI use adjusted for age, sex, time since transplantation. Model 2: Model 1 additionally adjusted for eGFR, deceased donor transplant, preemptive transplantation, primary renal disease. (DOCX) [file pmed.1003140.s006.docx]

**S3 Table**. Association of PPI use with graft failure in 703 stable KTRs.

|  |  | Graft failure | |
| --- | --- | --- | --- |
| Number of events |  | 110 | |
|  |  | HR (95%CI) | *P* |
| Crude |  | 1.20 (0.82 – 1.75) | 0.4 |
| Model 1 |  | 1.31 (0.87 – 1.97) | 0.2 |
| Model 2 |  | 1.10 (0.72 – 1.69) | 0.7 |

Model 1: PPI use adjusted for age, sex, BMI, time since transplantation. Model 2: Model 1 additionally adjusted for eGFR, proteinuria, deceased donor transplant, pre-emptive transplantation, primary renal disease.
