# Supplementary material for: The association between use of proton-pump inhibitors and excess mortality after kidney transplantation: A cohort study
Source: PLoS Med. 2020 Jun 15;17(6):e1003140. doi: 10.1371/journal.pmed.1003140 (PMC7295199; doi:10.1371/journal.pmed.1003140)
Supplement: S4 Table — Model 1: PPI use adjusted for time from baseline until follow-up. Model 2: Model 1 additionaly adjusted for age, sex, and BMI. (DOCX) [file pmed.1003140.s007.docx]

**S4 Table.** Association between PPI use and change in renal function during follow-up.

|  |  | Linear regression analysis | |
| --- | --- | --- | --- |
| Model |  | β (95%CI) | *P* |
| Crude |  | 0.75 (-1.82; 3.32) | 0.6 |
| Model 1 |  | 1.04 (-1.55; 3.62) | 0.4 |
| Model 2 |  | 0.10 (-2.45; 2.66) | 0.9 |

Model 1: PPI use adjusted for time from baseline until follow-up. Model 2: Model 1 additionaly adjustment for age, sex, and BMI.
