# Supplementary material for: The association between use of proton-pump inhibitors and excess mortality after kidney transplantation: A cohort study
Source: PLoS Med. 2020 Jun 15;17(6):e1003140. doi: 10.1371/journal.pmed.1003140 (PMC7295199; doi:10.1371/journal.pmed.1003140)
Supplement: S5 Table — Data are presented as mean ± SD, median with IQRs, or number with percentages (%). aMissing in 354 cases; bmissing in 299 cases. BMI, body mass index; eGFR, estimated glomerular filtration rate; HbA1c, hemoglobin A1c; HDL, high-density lipoprotein; IQR, interquartile range; LDL, low-density lipoprotein. (DOCX) [file pmed.1003140.s008.docx]

**S5 Table.** Baseline characteristics of 656 KTRs from the Leuven Renal Transplant Cohort.

| **Characteristics** | | | **Total population** |  | **Non-PPI users** |  | **PPI users** |  | ***P*** |
| --- | --- | --- | --- | --- | --- | --- | --- | --- | --- |
| Number of participants, *n (%)* | | | 656 (100) |  | 327 (49.8) |  | 329 (50.2) |  | n/a |
| Demographics | | |  |  |  |  |  |  |  |
|  | Age, *y* | | 55.0 ± 13 |  | 54 ± 13 |  | 57 ± 12 |  | 0.001 |
|  | Men, *n (%)* | | 394 (60.1) |  | 202 (61.8) |  | 192 (58.4) |  | 0.4 |
|  | Height, *cm* | | 170.3 ± 9.6 |  | 170.8 ± 9.7 |  | 169.9 ± 9.5 |  | 0.4 |
|  | BMI, *kg/m^2^* ^a^ | | 25.0 ± 4.7 |  | 25.0 ± 4.5 |  | 25.0 ± 4.9 |  | 0.9 |
|  | Diabetes mellitus, *n (%)* | | 110 (16.8) |  | 49 (15.0) |  | 61 (18.5) |  | 0.2 |
|  | Cardiovascular disease history, *n (%)* | | 152 (23.2) |  | 62 (19.0) |  | 90 (27.4) |  | 0.01 |
| Primary renal disease | | |  |  |  |  |  |  |  |
|  |  | Glomerulonephritis, *n (%)* | 123 (18.8) |  | 55 (16.8) |  | 68 (20.7) |  | 0.2 |
|  |  | Interstitial nephritis, *n (%)* | 79 (12.0) |  | 41 (12.5) |  | 38 (11.6) |  | 0.7 |
|  |  | Cystic kidney disease, *n (%)* | 127 (19.4) |  | 71 (21.47) |  | 56 (17.0) |  | 0.1 |
|  |  | Other congenital and hereditary kidney disease, *n (%)* | 31 (4.7) |  | 18 (5.5) |  | 13 (4.0) |  | 0.4 |
|  |  | Renal vascular disease, *n (%)* | 21 (3.2) |  | 10 (3.1) |  | 11(3.3) |  | 0.8 |
|  |  | Diabetes mellitus, *n (%)* | 57 (8.7) |  | 25 (7.6) |  | 32 (9.7) |  | 0.3 |
|  |  | Other multisystem diseases, *n (%)* | 37 (5.6) |  | 13 (4.0) |  | 24 (7.3) |  | 0.07 |
|  |  | Other, *n (%)* | 24 (3.7) |  | 10 (3.1) |  | 14 (4.3) |  | 0.4 |
|  |  | Unknown, *n (%)* | 157 (23.9) |  | 84 (25.7) |  | 73 (22.2) |  | 0.3 |
| Transplantation characteristics | | |  |  |  |  |  |  |  |
|  | Time since transplantation, *y* | | 3.0 (2.0 – 3.0) |  | 3.0 (2.0 – 3.0) |  | 3.0 (2.0 – 3.0) |  | 0.09 |
|  | Pre-emptive transplantation, *n (%)* | | 29 (4.4) |  | 17 (5.2) |  | 12 (3.6) |  | 0.3 |
|  | Deceased donor, *n (%)* | | 600 (91.5) |  | 294 (89.9) |  | 306 (93.0) |  | 0.2 |
| Renal function parameters | | |  |  |  |  |  |  |  |
|  | eGFR, *ml/min/1.73 m^2^* | | 50.1 ± 18.2 |  | 50.9 ± 17.5 |  | 49.3 ± 18.9 |  | 0.2 |
|  | Serum creatinine, *µmol/L* | | 128 (104 – 162) |  | 125 (104 – 158) |  | 130 (105 – 167) |  | 0.4 |
|  | Proteinuria (≥0.5 g/24h), *n (%) ^b^* | | 27 (7.6) |  | 11 (5.9) |  | 16 (9.3) |  | 0.2 |
| Hemodynamic parameters | | |  |  |  |  |  |  |  |
|  | Systolic blood pressure, *mmHg* | | 135 ± 17 |  | 134 ± 18 |  | 136 ± 16 |  | 0.4 |
|  | Diastolic blood pressure, *mmHg* | | 77 ± 11 |  | 77 ± 11 |  | 77 ± 11 |  | 0.8 |
|  | Heart rate, *bpm* | | 70 ± 10 |  | 70 ± 10 |  | 70 ± 11 |  | 0.9 |
| Laboratory parameters | | |  |  |  |  |  |  |  |
|  | Total cholesterol, *mmol/L* | | 4.59 ± 1.07 |  | 4.58 ± 0.89 |  | 4.59 ± 1.23 |  | 1.0 |
|  | HDL-cholesterol, *mmol/L* | | 1.48 ± 0.50 |  | 1.50 ± 0.49 |  | 1.47 ± 0.50 |  | 0.5 |
|  | LDL-cholesterol, *mmol/L* | | 2.40 ± 0.82 |  | 2.40 ± 0.69 |  | 2.41 ± 0.94 |  | 0.9 |
|  | Triglycerides, *mmol/L* | | 1.33 (1.05 – 1.91) |  | 1.27 (0.98 – 1.81) |  | 1.42 (1.07 – 1.97) |  | 0.007 |
|  | Glucose, *mmol/L* | | 5.5 (5.0 – 6.3) |  | 5.5 (5.10 – 6.11) |  | 5.6 (5.00 – 6.55) |  | 0.3 |
|  | HbA1c, *%* | | 5.9 (5.5 – 6.4 ) |  | 5.7 (5.5 – 6.1) |  | 6.0 (5.5 – 6.6) |  | 0.004 |
| Medication use | | |  |  |  |  |  |  |  |
|  | Antihypertensive drugs, *n (%)* | | 550 (83.8) |  | 269 (82.3) |  | 281 (85.4) |  | 0.3 |
|  | Platelet inhibitors*, n (%)* | | 227 (34.6) |  | 98 (30.0) |  | 129 (39.2) |  | 0.01 |
|  | Vitamin K antagonists, *n (%)* | | 23 (3.5) |  | 11 (3.4) |  | 12 (3.6) |  | 0.8 |
|  | Statins, *n (%)* | | 329 (50.2) |  | 154 (47.1) |  | 175 (53.2) |  | 0.1 |
|  | Proliferation inhibitors, *n (%)* | | 551 (84.0) |  | 273 (83.5) |  | 278 (84.5) |  | 0.7 |
|  | Calcineurin inhibitors,  *n (%)* | | 616 (93.9) |  | 305 (93.3) |  | 311 (94.5) |  | 0.5 |
|  | Prednisolone, *n (%)* | | 422 (64.3) |  | 185 (56.6) |  | 237 (72.0) |  | <0.001 |

Data are presented as mean ± SD, median (IQR) or number with percentages (%).^a^ missing in 354 cases; ^b^ missing in 299 cases. Abbreviations: BMI, body mass index; eGFR, estimated glomerular filtration rate; HbA1c, hemoglobin A1c; HDL, high-density lipoprotein, IQR, interquartile range; LDL, low-density lipoprotein.
