# Supplementary material for: The association between use of proton-pump inhibitors and excess mortality after kidney transplantation: A cohort study
Source: PLoS Med. 2020 Jun 15;17(6):e1003140. doi: 10.1371/journal.pmed.1003140 (PMC7295199; doi:10.1371/journal.pmed.1003140)
Supplement: S6 Table — Model 1: PPI use adjusted for age, sex, time since transplantation. Model 2: Model 1 additionally adjusted for eGFR, deceased donor transplant, preemptive transplantation, primary renal disease. (DOCX) [file pmed.1003140.s009.docx]

**S6 Table**. Association of PPI use with mortality in 656 stable KTRs from the Leuven Renal Transplant Cohort.

|  |  | All-Cause Mortality | |  |
| --- | --- | --- | --- | --- |
| No. events = 97 |  | HR | 95% CI | *P* |
| Crude |  | 2.47 | 1.61 – 3.78 | <0.001 |
| Model 1 |  | 2.06 | 1.34 – 3.16 | 0.001 |
| Model 2 |  | 1.75 | 1.12 – 2.73 | 0.01 |

Model 1: PPI use adjusted for age, sex, time since transplantation. Model 2: Model 1 additionally adjusted for eGFR, deceased donor transplant, pre-emptive transplantation, primary renal disease.
