# Supplementary material for: The association between use of proton-pump inhibitors and excess mortality after kidney transplantation: A cohort study
Source: PLoS Med. 2020 Jun 15;17(6):e1003140. doi: 10.1371/journal.pmed.1003140 (PMC7295199; doi:10.1371/journal.pmed.1003140)
Supplement: Attachment — Submitted filename: Response to the reviewers_final.docx [file pmed.1003140.s010.docx]

**Manuscript ID PMEDCINE-D-20-00132R2** “The Association between Use of Proton-Pump Inhibitors and Excess Mortality after Kidney Transplantation: A Prospective Cohort Study’’.

**Detailed, itemized response to the comments of the Editors**

***Editors***

*Comment 1: please revise to adhere to PLOS Medicine style; the colon should be followed by study descriptor only. We suggest you revise to “The association between use of Proton-Pump Inhibitors and Excess Mortality after Kidney Transplantation: A Prospective Cohort Study”*
**Response:** We thank the editors for having our manuscript reviewed and for providing us with suggestions to improve our manuscript. As suggested by the editors we have revised the manuscript title to the above mentioned suggestion: *“The association between use of Proton-pump inhibitors and Excess Mortality after Kidney Transplantation: A Prospective Cohort Study”*.

*Comment 2: Abstract- last sentence of the methods and findings section should be a limitation of your methodology*

**Response:** To accommodate the comment we have added the main limitations of the methodology to the abstract of the revised manuscript in lines 69-70 of the methods and findings section.

*Comment 3: Data availability- as per PLOS data policy, authors cannot be direct contacts for data requests. Please provide third party access, such as a data or ethics committee who can be contacted by interested authors.*

**Response:** We thank the editors for the comment and understand that according to the PLOS data policy, data have to be accessible for interested researches. To adhere to this policy we now provide an email address of the ethics committee of the University Hospitals Leuven, which can be contacted for data requests concerning the Leuven Renal Transplant Cohort. This e-mail address is [EC@UZLEUVEN.BE](mailto:EC@UZLEUVEN.BE) and is made available in the Data availability statement of the revised version of the manuscript.

*Comment 4: Author summary-At this stage, we ask that you include a short, non-technical Author Summary of your research to make findings accessible to a wide audience that includes both scientists and non-scientists. The Author Summary should immediately follow the Abstract in your revised manuscript. This text is subject to editorial change and should be distinct from the scientific abstract. Please see our author guidelines for more information:* [*https://journals.plos.org/plosmedicine/s/revising-your-manuscript#loc-author-summary*](https://journals.plos.org/plosmedicine/s/revising-your-manuscript#loc-author-summary)

**Response:** We thank the editors for the comment. Accordingly, we have provided an Author Summary of our research in the revised version of the manuscript on page 7 and 8.

*Comment 5: Throughout- please move the full stop before references to after, with a space in between text and brackets but not brackets and full stop. Please also provide square brackets for references. For example “ xxx [1].”*

**Response:** To accommodate the comment of the editor, we have changed the references style according to the above mentioned method throughout the revised version of the manuscript.

*Comment 6: Methods Line 136 – please provide details of the questionnaire used, citing it if previously published or providing a copy as supplementary information.*

**Response:** To accommodate the comment of the editor, we added the questionnaire about smoking behavior and alcohol use as supplementary information (S1 Appendix) to the revised version of the manuscript. We now refer to this supplemental questionnaire in line 184 of the methods section of the revised version of the manuscript.

*Comment 7: Discussion- please avoid assertions of primacy.*

**Response:** We thank the editors for the comment. Accordingly, we have removed assertions of primacy from the discussion section in line 377 and lines 446-448 of the revised version of the manuscript.

*Comment 8: Line 344-345 should contain “ a cause-effect relationship”.*

**Response:** We thank the editors for the comment. Accordingly, we have changed “cause-effects relationships” into “a cause-effect relationship” in line 458-459 of the revised version of the manuscript.

*Comment 9: Did your study have a prospective protocol or analysis plan? Please state this (eitherway) early in the Methods section.*

a) *If a prospective analysis plan (from your funding proposal, IRB or other ethics committee submission, study protocol, or other planning document written before analyzing the data) was used in designing the study, please include the relevant prospectively written document with your revised manuscript as a Supporting Information file to be published alongside your study, and cite it in the Methods section. A legend for this file should be included at the end of your manuscript.*
b) *If no such document exists, please make sure that the Methods section transparently describes when analyses were planned, and when/why any data-driven changes to analyses took place.*
c) *In either case, changes in the analysis—including those made in response to peer review comments—should be identified as such in the Methods section of the paper, with rationale.*

**Response:**

We thank the editors for the comment. For the current study, no separate prospective analyses plan was written before analyzing the data. The plan was made when we became aware of the study of Xie Y et al., published online July 4, 2017 in BMJ Open, in which an association between use of proton-pump inhibitors and increased risk of death was reported among Unites States Veterans. We realized that the data available in the TransplantLines Food and Nutrition Biobank and Cohort Study (clinicaltrials.gov identifier: NCT02811835), would allow us to prospectively investigate this in kidney transplant recipients, and to investigate whether a potential association would be independent of potential confounders. So, for the analyses we made use of existing data available in this prospective cohort study. However, the comment of the editors (and comment #1 of reviewer #3) makes us aware that the analyses that we performed were not predefined when setting up the cohort, so it would be appropriate to mention the current analyses as post-hoc analyses in a prospective cohort rather than prospectively defined analyses. Because in our cohort patients were included at different time points after transplantation, we decided that if we would find an association, even if it would be independent of time since transplantation, we would seek independent replication in a cohort with baseline measurements performed at a fixed time point in all subjects. While searching for this cohort, we investigated the association between PPI use and iron status (PMID: 31484461, published September 2019) and hypomagnesaemia (PMID: 31817776, published December 2019) in our cohort. After a while, we found out that the cohort from the University Hospitals Leuven could serve as independent replication cohort with baseline measurements performed at a fixed time point in all subjects and we proceeded with our manuscript on PPI use and mortality. No data-driven changes to analyses took place, other than those requested by the editors and the reviewers. To accommodate the comment of the editors (and comment #1 of reviewer #3), we now describe the study design in more detail in the methods section of the revised version of the manuscript (line 139, line 141-143 and line 151). Moreover, we describe changes in analysis made in response to reviewer comments in the methods section of the revised manuscript (lines 152-156, lines 228 – 234, lines 235-240 and lines 146-150 and lines 258-260.

*Comment 10: Please ensure that the study is reported according to the STROBE guideline, and include the completed checklist as Supporting Information. When completing the checklist, please use section and paragraph numbers, rather than page numbers. Please add the following statement, or similar, to the Methods: "This study is reported as per the Strengthening the Reporting of Observational Studies in Epidemiology (STROBE) guideline (S1 Checklist)."
Please report your study according to the relevant guideline, which can be found here: http://www.equator-network.org/
--> transparent reporting of a multivariable prediction model for individual prognosis or diagnosis (TRIPOD) guideline (S1 Checklist).*

**Response:** We thank the editors for the comment. Accordingly, we have completed the STROBE and TRIPOD checklists and added the above mentioned statement to the methods section of the revised version of the manuscript line 167-170. To adhere to the STROBE checklist, we now describe our primary and secondary outcomes in the materials and methods section of the revised manuscript (lines 152-155).

**Manuscript ID PMEDCINE-D-20-00132R2** “The Association between Use of Proton-Pump Inhibitors and Excess Mortality after Kidney Transplantation: A Prospective Cohort Study’’.

**Detailed, itemized response to the comments of the academic editor**

***Academic editor***

*Comment 1: As conceded by the authors, the group taking PPIs had a higher risk profile for mortality at baseline with multiple risk factors significantly more prevalent in the PPI group. There is therefore a significant risk of residual confounding. Moreover, the analysis did not take account of the indication for PPI prescription. Since conditions like peptic ulcer disease and gastro-oesophageal reflux (leading to Barrett’s oesophagus and oesophageal cancer) may increase the risk of death, there is also a risk of confounding by indication. This could be explored by conducting a subgroup analysis after excluding participants with these diagnoses or by comparing cause of death in those taking PPI versus not taking PPI. At very least this important limitation should be acknowledged in the paper.*

**Response:** We thank the academic editor for this comment and agree that it would be interesting to explore the risk of confounding by indication by comparing cause of death in those taking PPIs versus not taking PPIs. Accordingly, we compared cause of death in those taking PPIs versus those not taking PPIs and investigated whether use of PPIs was associated with cause-specific mortality (i.e. death due to cardiovascular diseases, infectious diseases, malignant diseases and miscellaneous causes). The majority of KTR died due to cardiovascular disease (37.1%), followed by death due to infectious diseases (24.2%), miscellaneous causes (20.1%), and malignant diseases (18.6%). PPI users more often died due to cardiovascular diseases compared to non-users (53 events vs. 19 events, log-rank *P*=0.001), and infectious diseases (32 events vs. 15 events, long-rank *P*=0.04). There were no significant differences in death due to malignant diseases and miscellaneous causes between PPI users and non-users (log-rank *P*=0.2 and log-rank *P=*0.3, respectively).

In univariable Cox regression analysis, we found that PPI use as compared to no PPI use was particularly associated with an increased risk of cardiovascular mortality (HR 2.42, 95%CI 1.43 – 4.08, *P*<0.001). This association remained independent of adjustment for potential confounders (Table S4). We also found that PPI use as compared to no PPI use was associated with increased mortality risk due to infectious diseases (HR 1.89, 95%CI 1.02 – 3.49, *P*=0.04). This association remained materially unchanged after adjustment for potential confounders, although it lost significance, mainly due to widening of the 95% confidence interval (HR 1.88, 95%CI 0.96 – 3.71, *P*=0.07, Table S4). Point estimates of hazard ratios of associations of PPI use as compared to no PPI use with malignant disease mortality and miscellaneous causes were lower and insignificant, both in univariable and multivariable Cox regression analyses (Table S4). It should be noted that results of these multivariable analyses are more prone to over-fitting because of the lower numbers of events for cause-specific mortality compared to all-cause mortality. To avoid the possibility of over-fitting as much as possible, we only tested the associations with cause-specific mortality using the first 2 models, in which associations are adjusted for a limited number of potential confounders (Table S4). To accommodate the comment of the academic editor (and comment #1 of reviewer #2) we added analyses on cause-specific mortality to the revised version of the manuscript and also describe that this was done in response to peer review comments in the methods sections of the revised manuscript. The methods of analyses investigating cause-specific mortality are described in lines 152-155, 159-164 and lines 245-250 of the methods section of the revised manuscript. The results of the analyses are described in lines 303-304 and line 326-328 of the results section of the revised manuscript. We furthermore briefly extended the discussion section of the revised version of the manuscript with mentioning of the results (line 403).

Although confounding by indication becomes less likely with results of these analyses, it cannot be excluded. Accordingly, we mentioning this in the limitations paragraph of the discussion section lines 468-472 of the revised version of the manuscript, by adding the following text: “Lastly, indications for PPI use such as peptic ulcer disease and gastroesophageal reflux disease may increase risk of death due to malignant diseases. Although confounding by indication becomes less likely with results of the cause-specific analyses, which did not demonstrate an increased mortality risk from malignant diseases, it cannot be excluded.”

**Manuscript ID PMEDCINE-D-20-00132R2** “The Association between Use of Proton-Pump Inhibitors and Excess Mortality after Kidney Transplantation: A Prospective Cohort Study’’.

**Detailed, itemized response to the comments of the Reviewers**

***Reviewer 1***

*Comment 1: I liked this paper. Very well thought out. Well controlled for distracting variables. We wrote on this.... "Concomitant Proton Pump Inhibitors with Mycophenolate Mofetil and the Risk of Rejection in Kidney Transplant Recipients. J. Knorr, M. Sjeime, L. Braitman, P. Jawa, R. Zaki, J. Ortiz. Transplantation March 15 2014. Vol 97 issue 5". Your team did not use. I like it. Well written. Good conclusion.*

**Response:** We thank the reviewer for the kind words. We have read the mentioned paper with great interest. To accommodate the comment of the reviewer (and comment #5 of reviewer #3), we performed additional analyses in which we investigated the potential association of PPI use with death-censored graft failure. During follow-up, a total of 110 KTR developed death-censored graft failure. We did not observe an association between PPI use and risk of graft failure (HR 1.20; 95%CI 0.82 – 1.75, *P*=0.4, Table S5). These results are in line with the results presented in the paper by Knorr J et al., which found no significant differences in incidence of graft failure between PPI users and non-users in the first year post transplantation (4.3% vs. 4.9%, *P*=0.8, Table 2 of the paper by Knorr et al. Transplantation 2014; 97: 518 - 24). To further accommodate the comment of the reviewer, we now mention the methods of the analyses investigating the association of PPI use with graft failure in line 246-248 of the revised version of the manuscript. The results from these analyses are described in lines 331-334 of the results section of the manuscript and Table S5 and the results are mentioned in the discussion section of the revised version of the manuscript, with citation of the manuscript by Knorr J et al. (lines 426 - 428).

***Reviewer2:***
*Comment 1: The investigators conducted this study to evaluate whether PPI use is associated with mortality risk in kidney transplant recipients. While this is important study, the findings of this study alone to evaluate mortality outcome might not adequately worth publishing in this high impact journal without significant modifications/revisions. There are many factors that can affect mortality risk in kidney transplant patients, such as cardiovascular, infection, rejection, allograft outcomes. However, the investigators barely look into this. It is possible that the investigators plan to separate these outcomes to several publications, since the investigators have published their works by using this cohort to evaluate several outcomes e.g. hypomagnesemia (PMID: 31817776) and iron status (PMID: 31484461). Without knowing the causes of mortality and lack of granularity (in addition to just 'mortality risk"), this finding of this study does not add much into the field of transplantation.*

**Response:** We thank the reviewer for the comment. To accommodate the comment of the reviewer (and comment #1 of the academic editor), we added data on cause-specific mortality and performed additional analyses in which we investigated the association of PPI use with cause-specific mortality. The majority of KTR died due to cardiovascular diseases (37.1%), followed by death due to infectious diseases (24.2%), miscellaneous causes (20.1%) and malignant diseases (18.6%). PPI users more often died due to cardiovascular diseases compared to non-users (53 events vs. 19 events, log-rank *P*=0.001), and infectious diseases (32 events vs. 15 events, long-rank *P*=0.04). There were no significant differences in death due to malignant diseases and miscellaneous causes between PPI users and non-users (log-rank *P*=0.2 and log-rank *P=*0.3, respectively).

In univariable Cox regression analysis, we found that PPI use as compared to no PPI use was particularly associated with an increased risk of cardiovascular mortality (HR 2.42, 95%CI 1.43 – 4.08, *P*<0.001), which remained independent of adjustment for potential confounders (Table S4). We also found that PPI use as compared to no PPI use was associated with increased mortality risk due to infectious diseases (HR 1.89, 95%CI 1.02 – 3.49, *P*=0.04). This association remained materially unchanged after adjustment for potential confounders, although it lost significance, mainly due to widening of the 95% confidence interval (HR 1.88, 95%CI 0.96 – 3.71, *P*=0.07, Table S4). Point estimates of hazard ratios of associations of PPI use as compared to no PPI use with malignant disease mortality and miscellaneous causes were lower and insignificant, both in univariable and multivariable Cox regression analyses (Table S4). It should be noted that results of these multivariable analyses are more prone to over-fitting because of the lower numbers of events for cause-specific mortality compared to all-cause mortality. To avoid the possibility of over-fitting as much as possible, we only tested the associations with cause-specific mortality using the first 2 models, in which associations are adjusted for a limited number of potential confounders (Table S4). To accommodate the comment of the reviewer (and comment #1 of the academic editor) we added analyses on cause-specific mortality to the revised version of the manuscript and also describe that this was done in response to peer review comments, in the methods sections of the revised manuscript. The methods of analyses investigating cause-specific mortality are described in lines 152-155, 159-164 and lines 245-250 of the methods section of the revised manuscript. The results of the analyses are described in lines 303-304 and line 326-328 of the results section of the revised manuscript. We furthermore briefly extended the discussion section of the revised version of the manuscript with mentioning of the results (line 403).

***Reviewer3****:*

*I applaud the authors on this significant work. This is an important study as it adds to the growing literature supporting an association between PPI and increased mortality, specifically involving kidney transplant recipients. The authors have provided two different cohorts demonstrating an association between PPI use and increased mortality. Based on current knowledge, I would not be surprised to find that such a positive correlation does exist between PPI and mortality. However, I question whether the reported risk is as great as presented as noted below.*

**Response:** We thank the reviewer for the kind words.

*Comment 1: Page 7, line 107. Looking at their prior described cohort (reference 28), this study appears to be a retrospective rather than prospective study as the prior cohort was established with an aim that would not have accrued the same information as presented in this study. If the authors can confirm this is accurate, the manuscript should be adjusted accordingly.*

**Response:** We appreciate the comment of the reviewer. The reviewer is right that the cohort was established with a different aim, but the information as presented in the current study, including information on PPI use was obtained during the baseline study visit and not retrospectively. Data regarding occurrence of death were prospectively obtained using the University Medical Center Groningen Renal Transplantation Database and were verified with the Dutch Civil Registration Office. So, for the analyses we made use of existing data available in this prospective cohort study, making the analyses prospective in nature. However, the comment of the reviewer (and comment #9 of the editor) makes us aware that the analyses that we performed were not predefined when setting up the cohort, so it would be appropriate to mention the current analyses as post-hoc analyses rather than prospectively defined analyses. To accommodate the comment of the reviewer (and comment #9 of the editor), we now describe the study design in more detail in the methods section of the revised version of the manuscript, with specific mentioning of the fact that it concerns a post-hoc analysis (line 139, line 141-143 and line 151).

*Comment 2: Page 7, line 112. What other KTR patients were excluded as noted for "concurrent systemic illnesses" aside from the named malignancies other than cured skin cancer, opportunistic infections, and overt congestive heart failure?*

*It should be noted that this study may not be generalizable to KTR patients who had these systemic illnesses. As opportunistic infections can be an acute process rather than chronic, it would have been helpful to include these patients. In addition, it would be interesting to know the results from patients with overt CHF as these patients may have an increased atherosclerotic burden compared to non-CHF patients. As such, these patients.*

**Response:** We thank the reviewer this comment. The comment of the reviewer makes us aware of the fact that we erroneously used the word excluded rather than the word ineligible, which would have been more appropriate. The word excluded could raise the suggestion that there are data from patients which have been excluded. However, for building the cohort, KTR with concurrent systemic illnesses, including malignancies other than cured skin cancer, opportunistic infections, and overt congestive heart failure were considered ineligible to participate. So, the TransplantLines Food and Nutrition Biobank and Cohort Study does not contain data on subjects which have been excluded. To accommodate the comment of the reviewer, we changed the word “excluded” into “not considered eligible for the study” in line 147. We agree with the reviewer that for the current analyses, it would have been helpful if we would have included patients with opportunistic infections and patients with overt congestive heart failure, but due to the prospective design of the study, with baseline measurements that have been performed in the past, we are unable to include such patients. To further accommodate the comment of the reviewer we also added mentioning of the fact that we did not include patients with opportunistic infections and patients with overt heart failure to the limitations paragraph of the discussion section of the revised version of the manuscript (lines 456-458).

*Comment 3: The patients in the non-PPI group had a significantly increased time since transplantation compared to the PPI group. This would seem to cause a significant survival selection bias. This may be due to the retrospective nature (see above regarding question of retrospective vs prospective) of this study. If this was a prospective study as the authors stated, then performing a study where patients were at the same timepoint after kidney transplant with or without PPI would have helped avoid this significant survival bias (e.g. picking recipients who received their kidney transplant a certain year and then evaluating their mortality outcomes based on PPI vs non-PPI). I appreciate that the authors adjusted for this factor, but as we know, there are many unknown confounders that may be present in an individual who has survived with a functional graft already for 9 years vs an individual for 4 years.*

**Response:** We agree with the reviewer that although we adjusted for time since transplantation, survival bias cannot be excluded in the analyses performed using data of the TransplantLines Food and Nutrition Biobank and Cohort Study. For this reason, we sought for independent replication in a cohort with baseline measurements performed at a fixed time point in all subjects. Such data were available in the cohort from the University Hospitals Leuven. In this replication cohort, KTR were included at a median of 3.0 years after transplantation, with no significant differences in time since transplantation between the PPI group and the non-PPI group at baseline (Table S2). Prospective analyses in this replication cohort showed similar results as analyses from the TransplantLines Food and Nutrition Biobank and Cohort Study (see Table S3). To accommodate the comment of the reviewer, we now describe the reasons why we sought for independent replication in an independent cohort in more detail in the methods section of the revised manuscript (lines 266-270). To further accommodate the comment of the reviewer we discuss the possibility of survival bias in the primary cohort in the limitations section of the revised manuscript (lines 464-468).

*Comment 4: Table 1: It would have been interesting to include antiplatelet use. Previously, there was some concern that PPI's may interfere with clopidogrel efficacy, and KTR are at high risk for CV complications and mortality. Furthermore, since patients on dual antiplatelet medications for coronary atherosclerosis either before or after stenting tend to also be on PPI's, these patients are likely at higher risk for CV complications and mortality. The omission of patients with overt heart failure and not adjusting for antiplatelet agents may influence the results of this study.*

**Response:** We thank the reviewer for the suggestion and agree that it would be interesting to adjust for the use of platelet inhibitors. To accommodate the comment of the reviewer, we splitted up the data on use of anticoagulants presented in Table 1 into use of antiplatelet agents and use of vitamin K antagonists and added these data to Table 1 of the revised version of the manuscript. A total of 144 (20.5%) non-PPI users versus 96 (24.1%) PPI users were on antiplatelet agents (P=0.006). Of these, 1 PPI non-user (0.3%) versus 6 (1.5%) PPI users were on dual antiplatelet medication (*P*=0.2). We added results of these analyses to the results section of the revised version of the manuscript (line 298-299). To further accommodate the comment of the reviewer, we repeated all analyses in which we previously adjusted for anticoagulant use, with adjustment for both use of platelet inhibitors and use of vitamin K antagonists as separate covariates. In these analyses, the association between PPI use and mortality remained materially unchanged (HR 1.49, 95% CI 1.07 – 2.09, *P*=0.02; see Table 2 of the revised version of the manuscript). Methods of this analysis are described in line 223 of the revised version of the manuscript and results of this analysis are demonstrated in line 313-314 and Table 2 of the revised version of the manuscript.

*Comment 5: Page 11, line 220. What percentage of patients from the PPI vs non-PPI group developed graft failure? Along this line, was the eGFR was only determined at the initial baseline measurement or was it determined closer to patient death? Since tubulointerstitial nephritis (acute or chronic) is associated with PPI's, it would have been useful to see if PPI patients also had a decreased eGFR at time of death. Since renal function is associated with mortality, this would be another important covariate to control for (aside from what the authors had already done by omitting patients who developed graft failure.*

**Response:** We thank the reviewer for the suggestion to also investigate the potential association of PPI use with graft failure. To accommodate the comment of the reviewer, we added data on death-censored graft failure to the revised version of the manuscript. During follow-up, a total of 110 KTR developed death-censored graft failure. There was no significant difference in graft failure development between PPI users compared to PPI non-users (64 events (58.2%) vs. 46 events (41.8%), log-rank *P*=0.4). Cox-proportional hazard analyses in which we adjusted for potential confounders yielded similar results, with absence of an association (see Table S5 of the revised version of the manuscript). The methods of the analyses investigating the association of PPI use with graft failure are described in line 245-248 of the revised version of the manuscript. The results from these analyses are described in lines 301-302 of the results section of the manuscript and Table S5. Accordingly, we now discuss these results in the discussion section of the revised manuscript (lines 426 - 428).

To further accommodate the comment of the reviewer we also obtained information on the last measurement of serum creatinine before occurrence of either death, death-censored graft failure or end of follow-up. These levels were used to calculate eGFR at the moment closest to patient death, as requested by the reviewer. For these eGFR data, median follow-up after baseline measurement was 6.5 [4.6 – 8.6] years (with this longer time between baseline and follow-up being the consequence of extension of follow-up time to 31 December 2018 (in response to comment #2 of reviewer #4)). Mean change in renal function over this period was -7.13 ± 17.1 ml/min/1.73m^2^. In a crude linear regression analysis, PPI use was not associated with eGFR decline (β= 0.75, 95% CI -1.81; 3.32, *P*=0.6). Results remained materially unchanged when we adjusted for time from baseline to follow-up (β= 1.04, 95%CI -1.55; 3.62, *P*=0.4). Additional adjustment for age, sex and BMI did not materially alter the association (β= 0.10, 95%CI -2.45; 2.66, *P*=0.9). To further accommodate the comment of the reviewer, we performed sensitivity analyses in which we adjusted for eGFR change cumulative to already existing adjustments performed in model 2 of the Cox regression analyses. Results remained unchanged when we adjusted for change in eGFR (HR 1.68, 95% CI 1.22 – 2.33, *P*=0.002). The methods of these analyses are described in lines 250 -260 of the revised manuscript. The results are described in lines 335 - 342 and in Table S6 of the revised version of the manuscript.

*Comment 6: Table 2 and Table 3: Was there a model where you adjusted for all of the models (1-6) combined? What was this result? I appreciate the authors providing several mechanisms for PPI use and increased mortality.*

**Response:** We thank the reviewer for the question and for the kind words. The comment of the reviewer makes us realize that in the manuscript we did not made reference to the generally accepted rule of thumb regarding the number of events required per each covariate to be adjusted for in Cox regression analyses, to avoid occurrence of over-fitting and overadjustment (Concati J et al. J Clin Epidemiol 1995; 48: 1495-1501, Peduzzi P et al. J Clin Epidemiol 1995; 48: 1503-1510). Because of this rule of thumb, which allows for 1 covariate per approximately 10 events, with 123 events available for analyses, we limited the number of covariates per model to a maximum of 13 per model. To adhere to this maximum amount and to be able to test for as many as possible potential confounding covariates, we each time separately performed adjustments in addition to adjustments performed in model 2. To accommodate the comment of the reviewer, we now added mentioning of this to the materials and methods section of the revised version of the manuscript (lines 218-220). Of note, to accommodate comment #2 of reviewer #4, follow-up for all prospective analyses was extended until 31 December 2018, which resulted in an increase of the number of events to 194, which allows for inclusion of 19 to 20 variables in one model. To further accommodate the comment of the reviewer we also performed analyses in which we adjusted for all covariates in one cumulative model, while avoiding occurrence of overadjustment bias due to inclusion of overlapping variables within one biological domain in the same statistical model and for factors which lie in the causal pathway (Schisterman EF et al. Epidemiology 2009; 20:488-495, Patorno E et al. Drug Saf 2015; 38: 295-310). In our analyses, this could be the case if we would simultaneously adjust for diabetic nephropathy together with diabetes, and for use of vitamin K antagonists, platelet inhibitors, and use of anti-hypertensive drugs together with cardiovascular disease history. Also, magnesium and iron could lie in the causal pathway. In a final model in which we adjusted for all variables of models 1-5 combined, except diabetic nephropathy, vitamin K antagonists, platelet inhibitors, and use of anti-hypertensive drugs, the association between PPI use and all-cause mortality remained (HR 1.44, 95% CI 1.03 – 2.02, *P*=0.03). Alternatively, the association between PPI use and all-cause mortality remained in a final model in which we adjusted for all variables of models 1-5 combined, except diabetes and cardiovascular disease history (HR 1.48, 95% CI 1.05 – 2.07, *P*=0.02). Accordingly, in the materials and methods section of the revised version of the manuscript, we now added referencing to Concati J et al. J Clin Epidemiol 1995; 48: 1495-1501, Peduzzi P et al. J Clin Epidemiol 1995; 48: 1503-1510 and to Schisterman EF et al. Epidemiology 2009; 20:488-495, Patorno E et al. Drug Saf 2015; 38: 295-310. We moreover added mentioning of the analyses in which all variables were combined with avoidance of inclusion of overlapping variables in one domain (lines 228-234). Results of this analysis are described in lines 317-332 of the revised manuscript.

***Reviewer 4:***

*The authors have attempted to investigate the effect of PPI therapy on mortality in a large single center cohort of stable outpatient KTR, and performed independent replication of the results in a second cohort.*

*Comment 1: Cohort demographics and characteristics - is the sample representative of wider population? It is noted from the discussion on limitations that subjects were predominantly Caucasian - what other comparisons can be made to better understand generalisability of the results? It is also noted that the data used is from a previously described cohort, but a brief overview or summary within this manuscript would be helpful to the reader in their assessment of how robust the findings and conclusions are. Confounding has been accounted for adequately, given the limitations of the data, and the description, production and presentation of all 6 models is very useful.*

**Response:** We thank the reviewer for the comment and for the words of appreciation. The comment of the reviewer (and comment #2 of reviewer #3) makes us realize that next to the fact that our population mainly consisted of Western-European Caucasians patients, patients with concurrent systemic illnesses, including malignancies other than cured skin cancer, opportunistic infections, and overt congestive heart failure were considered ineligible to participate in the TransplantLines Food and Nutrition Biobank and Cohort Study. To accommodate the comment of the reviewer (and comment #2 of reviewer #3), we now describe this more clearly in the materials and methods section of the revised version of the manuscript and discuss this in the limitations paragraph of the discussion section of the revised version of the manuscript (lines 455-458).

To further accommodate the comment of the reviewer (and comment #9 of the editor) we now describe the study design in more detail in the methods section of the revised version of the manuscript (line 139, line 141-143 and line 151).

*Comment 2: Under methods: "Follow-up was recorded until September 2015. " Can follow-up now be extended? The current data has median follow-up of 5.3 [4.5-6.0] years, which could almost be doubled in length adding great value to the longitudinal aspects of this study.*

**Response:** We thank the reviewer for the comment. To accommodate the comment of the reviewer, we extended follow-up up to 31 December 2018. Accordingly, we repeated all prospective analyses using the extended follow-up data and modified the revised version of the manuscript in line with these data and analyses. During a median follow-up of 8.2 [4.7 – 9.0] years, 194 KTR died with a functioning graft. Out of the 398 KTR who used PPIs at baseline, 132 (33.2%) died during follow-up, whereas out of the 305 KTR who did not use PPIs at baseline, 62 (20.3%) died during follow-up (log-rank *P*<0.001, Figure 1 of the revised manuscript). The association between PPI use and mortality remained materially unchanged when performing Cox regression analyses using the extended follow-up data (HR 1.86, 95%CI 1.38 – 2.52, *P*<0.001), and independent of adjustment for potential confounders as shown in Table 2 of the revised version of the manuscript. The extension of the follow-up did also not result in material changes in results of dose response analyses (see Table 3 of the revised version of the manuscript). To further accommodate the comment of the reviewer, we now mention that we extended the follow-up of this study in line 156-157 of methods section of the revised manuscript. The results of the updated analyses are described in lines 301-317 of the results section of the revised manuscript. Changes in results from the dose response analysis are described in line 350-351 of the revised manuscript and in Table 3. Furthermore, we updated the Kaplan-Meier survival curve using the extended follow-up data (Figure 1 of the revised version of the manuscript).

*Comment 4: The statistical techniques appear to be appropriate for the data and the research question in hand. It is also good to see that a sensitivity analysis of missing data has been performed, by comparing mulitple imputation results to no imputation. Did the authors undertake any sub-group anaylses (by which, I am not referring to the dose-response analysis shown in Table 3)? Was there an association between outcome and age, for instance, as suggested by the literature [23-25]?*

**Response:** We thank the reviewer for the kind words and the question about sub-group analyses. Sub-group analyses should be based on evidence for presence of effect-modification as indicated by finding of significant interaction. There was indeed a significant association between outcome and age (HR 1.08, 95%CI 1.06-1.09 , P<0.001), but there was no significant interaction by age (P=0.8 for interaction). To accommodate the comment of the reviewer, we additionally performed analyses for potential effect modification by sex, eGFR, diabetes, and medication for which significant baseline differences were present, including antihypertensive drugs, platelet inhibitors, vitamin K antagonists and calcineurin inhibitors. This was tested by adding interaction terms consisting of PPI use and the variable of interest to model 2 of the Cox proportional hazard regression analyses. There were no significant interactions between PPI use and age (*P*_interaction_=0.8), sex (*P*_interaction_=0.6), eGFR (*P*_interaction_=0.09) and diabetes (*P*_interaction_=0.2) for the association with mortality. In addition, no effect modification was observed between PPI use and the use of antihypertensive drugs, platelet inhibitors, vitamin K antagonists and calicineurin inhibitors (*P*_interaction_=0.3, *P*_Interaction_=0.2, *P*_interaction_=0.9, *P*_interaction_=0.9, respectively). The methods of these analyses are described in the methods section of the revised manuscript (line 235-240). The results of these analyses are described in the results section of the revised version of the manuscript (lines 323 - 326).

Comment 5: *The conclusions are fair given the study design undertaken, and the manuscript as a whole is well written, clear and concise. The Tables and Figures are also clear and informative.*

**Response:** We thank the reviewer for the kind words.
