# Supplementary material for: The association between use of proton-pump inhibitors and excess mortality after kidney transplantation: A cohort study
Source: PLoS Med. 2020 Jun 15;17(6):e1003140. doi: 10.1371/journal.pmed.1003140 (PMC7295199; doi:10.1371/journal.pmed.1003140)
Supplement: Attachment — Submitted filename: Response to the reviewers_R3_final.docx [file pmed.1003140.s011.docx]

**Manuscript ID PMEDCINE-D-20-00132R3** “The Association between Use of Proton-Pump Inhibitors and Excess Mortality after Kidney Transplantation: A Cohort Study’’.

**Detailed, itemized response to the comments of the Editors**

***Editors***

*Comment 1: Title- please remove the word “prospective” since the analyses were carried out after the cohort had already been established*

**Response:** We thank the editors for their time and effort spent on reviewing our manuscript and for expressing the intention to accept the manuscript for publication when all remaining editorial issues have been addressed. As suggested by the editors we have removed the word “prospective” from the manuscript title, so that it now reads: *“The association between use of Proton-pump inhibitors and Excess Mortality after Kidney Transplantation: A Cohort Study”*.

*Comment 2: Author summary; Line 82-please revise to “in the long term”*

**Response:** We thank the editors for noticing this grammatical error. Accordingly, we changed “on the long term” to “in the long term” in line 68 of the author summary of the revised manuscript.

*Comment 3:* *Introduction;* Line 117- could you rephrase “gastrointestinal complaints” for specificity?

**Response:** To accommodate the comment of the editors, we rephrased “gastrointestinal complaints” into “dyspeptic symptoms” in line 103 of the revised manuscript.

*Comment 4:* *Methods; Please provide the information about your replication cohort earlier in the methods section within 2.1- Design and Study population. Please also remove lines 265-269 as these are better placed in introduction/discussion sections.*

**Response:** Accordingly, we moved the paragraph about the replication cohort to the methods section paragraph 2.1- Design and Study population lines 158 – 170 of the revised manuscript. Moreover, as suggested by the editors we removed lines 265-296 about the rationale for the use of a replication study and added this rationale to the introduction section of the revised manuscript (lines 121 - 125).

*Comment 5: Results; Line 300-301 do you mean “excluded from analyses” instead of “censored”?*

**Response:** We thank the editors for noting this unclearness. Re-reading the sentence, we noticed that our use of the words “were censored from analyses”, indeed may make the impression that they were excluded from analyses, which is not the case, because by censoring them at the time of graft failure, they contributed to analyses until time of graft failure. Accordingly, we removed the words “from analyses” after the words “were censored” and replaced them by “at time of graft failure” in line 315 in the results section of the revised manuscript, so that the concerned subsentence now reads as “were censored at time of graft failure”.

*Comment 6: In the Kaplan-Meier curve in Figures 1 and 2, please provide the number at risk for each time interval.*

**Response:** We thank the editors for the comment. Accordingly, we now provide the number at risk for each time interval in both Kaplan-Meier curves in the revised version of the manuscript (Fig 1 and Fig 2).

**Manuscript ID PMEDCINE-D-20-00132R3** “The Association between Use of Proton-Pump Inhibitors and Excess Mortality after Kidney Transplantation: A Cohort Study’’.

**Detailed, itemized response to the comments of the Reviewers**

***Reviewer #2***

*Comment 1: My major concerns still persist. Patients who received PPI usually are different than those who received PPI. Those on PPI usually have high cardiovascular disease and on antiplatelet agents and/or anticoagulations. Medications such as aspirin, other antiplatelets/anticoagulation should be taken into consideration.*

**Response:** We thank the reviewer for the time and effort spent on reviewing our manuscript. We sincerely apologize if it may not have been clear from our former rebuttal letter and from the former revised version of the manuscript (R2) that data on use of antiplatelet agents and use of vitamin K antagonist were added to Table 1 of the revised version of the manuscript (R2), which has been retained in the current revised version of the manuscript (R3). Also as part of the former revision, we replaced all analyses in which we previously adjusted for anticoagulant use as a single covariate, with adjustment for both use of platelet inhibitors and use of vitamin K antagonists as separate covariates. In these analyses, the association between PPI use and mortality remained materially unchanged (HR 1.49, 95% CI 1.07 – 2.09, *P*=0.02; see Table 2 of the former revised version of the manuscript (R2), which has been retained in the current revised version of the manuscript (R3)). As part of the previous rebuttal, we also described the methods of this analysis in line 221-222 of the former revised version of the manuscript and results of this analysis are described in lines 295-296 and Table 2 of the former revised version of the manuscript (R2). In the current revised version of the manuscript (R3), these respective line numbers are 229 -230 and 325 -327.

*Comment 2:*

*Also, more data on transplant-related are needed. Data on induction therapy, and HLA-mismatch, and DSA, and KDPI, which are important data for kidney transplant studies, have not been included and adjusted.*

**Response:** We thank the reviewer for the suggestion to add more transplant-related data to the manuscript. To accommodate the comment of the reviewer, we added available data on the kidney donor profile index (KDPI), including donor age, donor weight, donor height, donor serum creatinine and number of HLA-mismatches and data on donor sex and applied induction therapy to Table 1 of the revised version of the manuscript (R3). Unfortunately, since the kidney donor profile index (KDPI) has been developed for application in circumstances of transplantation with kidneys derived from deceased kidney donors, while more than 30% of the kidneys in our population were derived from living donors (Rao et al. Transplantation 2009; 88: 231-236 and OPTN guideline: A Guide to Calculating and Interpreting the Kidney Donor Profile Index; May 15 2009 ), the KDPI cannot be used in our setting. To further accommodate the comment of the reviewer, we performed adjustment for the available donor characteristics and immunological parameters in Cox regression analyses, in a separate Cox model. In this separate model, we adjusted for donor age, donor sex, donor weight, donor height, donor serum creatinine, total HLA-mismatches and applied induction therapy, in addition to adjustments performed in model 2 (Model 3, Table 2 of the revised version of the manuscript (R3)). In this additional model, the association between PPI use and mortality remained materially unchanged (HR 1.67, 95%CI 1.19 -2.34, *P*=0.003, Table 2). Furthermore, the same model was added to adjustments performed for the dose-response analyses. These adjustments did also not materially affect the association (Model 3, Table 3). Lastly, we also adjusted for donor characteristics and immunological factors in the cumulative models in which we adjusted for all variables of model 1-6 combined. Also in these analyses, the association between PPI use and all-cause mortality remained materially unchanged. Methods of these analyses are described in the statistical analyses section of the revised version of the manuscript (lines 225 -228 and line 236) with specific mentioning of the fact that this model was added in response to a comment of one of the reviewers. Results from these analyses are described in line 326 and lines 329 – 336 of the results section of the revised version of the manuscript.

Unfortunately, data on donor specific antigens (DSAs) were not available in our cohort and it was therefore not possible to adjust for this potential confounder in our Cox regression analyses. To further accommodate the comment of the reviewer we mention this as a limitation in the discussion section of the revised manuscript (lines 503 - 504).

*Comment 3: Causes of death have not been look at. PPI per se does not cause mortality, but the differences in characteristics, commodities, that cause mortality. Rejection as outcome also has not been demonstrated in this study.*

**Response:** We thank the reviewer for the comment. Again, we sincerely apologize if it may not have been clear from our former rebuttal letter and former revision of the manuscript (R2) that we added data on cause-specific mortality to the revised version of the manuscript as suggested by reviewer #2 of the former revision round. In response to comments of reviewer #2 of the former revision round and the academic editor, we added data on cause-specific mortality and performed additional analyses in which we investigated the association of PPI use with cause-specific mortality. In the former revised version of the manuscript (R2) and the former detailed itemized response to comments of the reviewers, it can be found that the majority of KTR died due to cardiovascular diseases (37.1%), followed by death due to infectious diseases (24.2%), miscellaneous causes (20.1%) and malignant diseases (18.6%). PPI users more often died due to cardiovascular diseases compared to non-users (53 events vs. 19 events, log-rank *P*=0.001), and infectious diseases (32 events vs. 15 events, long-rank *P*=0.04). There were no significant differences in death due to malignant diseases and miscellaneous causes between PPI users and non-users (log-rank *P*=0.2 and log-rank *P=*0.3, respectively). These changes were retained in the current revised version of the manuscript (R3).

In univariable Cox regression analysis, we found that PPI use as compared to no PPI use was particularly associated with an increased risk of cardiovascular mortality (HR 2.42, 95%CI 1.43 – 4.08, *P*<0.001), which remained independent of adjustment for potential confounders (this was added as Table S4 to the former revised version of the manuscript (R2). These changes were also retained in the current revised version of the manuscript (R3)). We also found that PPI use as compared to no PPI use was associated with increased mortality risk due to infectious diseases (HR 1.89, 95%CI 1.02 – 3.49, *P*=0.04). This association remained materially unchanged after adjustment for potential confounders, although it lost significance, mainly due to widening of the 95% confidence interval (HR 1.88, 95%CI 0.96 – 3.71, *P*=0.07, also see Table S4 of the former revised version of the manuscript (R2). These changes were also retained in the current revised version of the manuscript (R3)). Point estimates of hazard ratios of associations of PPI use as compared to no PPI use with malignant disease mortality and miscellaneous causes were lower and insignificant, both in univariable and multivariable Cox regression analyses (also see Table S4 of the former revised version of the manuscript (R2). These changes were also retained in the current revised version of the manuscript (R3)).

To further accommodate the comment of the reviewer, we also investigated whether PPI use is associated with an increased risk of biopsy proven acute rejection (BPAR). During follow-up of 8.2 years, 13 KTR developed BPAR. In crude Cox regression analysis, PPI use was not significantly associated with BPAR (HR 1.73, 95% CI 0.53 – 5.61, *P*=0.4). Because of the low number of events, there is no realistic possibility to adjust for potential confounders. Therefore, no meaningful conclusions can be drawn regarding the association of PPI use with risk of BPRAR. To further accommodate the comment of the reviewer we now describe this analysis in the materials end methods section (line 255-256) and in the results section (lines 348 - 351) of the revised version of the manuscript.

***Reviewer #4***

*Comment 1: The authors have responded to each comment in turn, adding data, completing further analyses, and amending the manuscript contents accordingly.*

**Response:** We thank the reviewer for his time and effort spent in reviewing our revised manuscript and for the words of appreciation.

***Production Issues***

FIGURES and GRAPHICS:

*1. Please ensure that all main figure files are cited in ascending numerical order in the main text of the article.*

**Response:** All figures (Fig 1 and Fig 2) are cited in ascending numerical order in the main text of the article.

*2. Please ensure that all main figures are referenced in the manuscript as Fig 1, Fig 2, etc. (including capitalization), rather than Figure 1, Figure 2, etc. Please note, however, that the file names themselves must not include the space, i.e. Fig1.tif, Fig2.tif, etc.*

**Response:** We now apply the correct reference method for our figures (Fig 1 and Fig 2). File names do not include spaces.

*3. Please ensure that all main figure legends are placed after the paragraph containing their first in-text citation.*

**Response:** All figure legends are placed after the paragraph containing their first in-text citation.

*4. Please note that your figures do not currently comply with our publication requirements. For further instructions to assist with your Figure revisions, please refer to our Figure Guidelines at:*

[*https://eur03.safelinks.protection.outlook.com/?url=http%3A%2F%2Fjournals.plos.org%2Fplosbiology%2Fs%2Ffigures&amp;data=02%7C01%7Cr.m.douwes%40umcg.nl%7C3c1fd2b5a0244c4aa07008d7ec458e5d%7C335122f9d4f44d67a2fccd6dc20dde70%7C0%7C0%7C637237655802163209&amp;sdata=Xbdeqj563JsYVNa%2FgAk9O1d3HQDgYKTNh8kd6PGvfVM%3D&amp;reserved=0*](https://eur03.safelinks.protection.outlook.com/?url=http%3A%2F%2Fjournals.plos.org%2Fplosbiology%2Fs%2Ffigures&amp;data=02%7C01%7Cr.m.douwes%40umcg.nl%7C3c1fd2b5a0244c4aa07008d7ec458e5d%7C335122f9d4f44d67a2fccd6dc20dde70%7C0%7C0%7C637237655802163209&amp;sdata=Xbdeqj563JsYVNa%2FgAk9O1d3HQDgYKTNh8kd6PGvfVM%3D&amp;reserved=0)

If you need assistance, please contact us directly at [graphics@jjeditorial.com](mailto:graphics@jjeditorial.com) so we can further help with this matter. Please send any relevant source files and tell us what application(s) and what operating system you are using**.**

*4a) Please resave your Figures 1 and 2 using LZW compression (loss-less) in order to reduce the file size by over 75% and re-upload.*

*In Photoshop: File→Save As. Save as TIFF, Image Compression set to LZW, Pixel Order set to Interleaved, Byte Order set to IBM PC.*

*In GIMP: File→Save As. Click the + sign next to Select File Type (By Extension). From the menu that appears, select TIFF. Click Save. Set Compression to LZW. If you're prompted about layers in the file, select Flatten Image.*

**Response:** We thank the publication manager for the clear instructions. Accordingly, we resaved our figures using LZW compression, pixel order set to interleaved, byte order set to IBM PC.

*4b) Figures 1 and 2 have been submitted in a layered TIF file format which we don’t accept for figures. To ensure that your figures publish properly, please flatten the layers in each figure file (Layer→Flatten Image in Photoshop/GIMP) and re-upload. Make sure to save your TIF files with LZW Compression turned on.*

**Response:** Accordingly, we have flattened the layers in each figure file using the above instructions.

5) Please ensure that all main figure files follow PLOS naming conventions in the File Inventory. The file name should be in the format “Fig1.tif” and the file description should be “Fig 1”.

**Response:** Accordingly, our files are now renamed: “Fig1.tif” and “Fig2.tif”.

TABLES:

*1. Please cite main tables in ascending numerical order in your main article file.*

**Response:** Tables are cited in numerical order in our main article file.

*2. Please ensure that all main tables in their entirety (including titles and footnotes), are placed after the paragraph containing their first in-text citation.*

**Response:** All main tables are now placed after the paragraph containing their first in-text citation.

*3. Table 1 uses returns to control text wrapping. This is indicated by a [¶ or ↵] symbol when "Show Paragraph Marks" is turned on in Word. Please either split those cells into separate cells to achieve alignment or remove the returns.*

**Response:** We removed the returns before the headers in each column accordingly.

METADATA:

*1. Please ensure that the funders and grant numbers match between the Financial Disclosure field and the Funding Information tab in your submission form. Note that the funders must be provided in the same order in both places as well.*

**Response:** All funders and grant numbers are matched and provided in the same order in both the financial disclosure field and the funding information tab.

*2. In accordance with PLOS' data policy, please ensure that your Data Availability Statement in the submission form clearly identifies how readers can access your data. Data cannot be available on request, or only accessible by contacting one of the authors. Note that your Data Availability Statement will be typeset as it is written, so please ensure it is in complete sentences and appears as you would like it to in the published version.*

**Response:** We provided a data availability statement which clearly identifies how readers can access our data.

For details on our data policy, please see

<https://eur03.safelinks.protection.outlook.com/?url=http%3A%2F%2Fjournals.plos.org%2Fplosmedicine%2Fs%2Fdata-availability&amp;data=02%7C01%7Cr.m.douwes%40umcg.nl%7C3c1fd2b5a0244c4aa07008d7ec458e5d%7C335122f9d4f44d67a2fccd6dc20dde70%7C0%7C0%7C637237655802163209&amp;sdata=f2ad4h4hkjOdiELoiR8nFRu5gzgVW%2FDCoe8bPA156pU%3D&amp;reserved=0>

ARTICLE FILE:

*1. Please indicate the corresponding author in the byline by placing an asterisk (*) after their affiliation number.*

**Response:** Accordingly, we now indicate the corresponding author with an asterisk after the affiliation number.

*2. Please include the corresponding author's email address on the title page of your manuscript, indicated by an asterisk (*). Only an asterisk and the email address itself are required.*

**Response:** The email address of the corresponding author is provided and indicated by an asterisk.

*3. The name for your co-author Stephan S.J.L. Bakker does not match across the article file and the submission form. Please ensure that all author names match verbatim, including the use of a middle name/initials and any special characters (accents, umlauts, etc.), as correct spelling and formatting of author names is vital for accurate indexing in PubMed.*

**Response:** Accordingly, we now changed “Stephan S.J.L Bakker” to the correct name“ Stephan J.L. Bakker” in the submission form.

*4. Please remove the funding information from your manuscript file. The published version is pulled automatically from the “Financial Disclosure” field in the submission form and must not be duplicated. Please therefore take this opportunity to check that the information in this field is correct and completed in full.*

**Response:** We have removed the funding information from the manuscript file and checked whether the statement in the submission form was complete.

*5. Please remove the Competing Interests section from your manuscript file. The published version is pulled automatically from the "Competing Interests" field in the submission form and must not be duplicated. Please therefore take this opportunity to check that the information in this field is correct and completed in full.*

**Response:** We have removed the competing interest information from the manuscript file and checked the submission form.

6. Please remove your Author Contributions section from your manuscript file as this information is automatically pulled from the authors section of our submission form and must not be duplicated. Please therefore take this opportunity to check that the information in this field is correct and completed in full.

**Response:** We we have removed the author contributions section from the manuscript file and checked the submission form.

7. Please ensure that your Ethics statement is available in the Methods section of your manuscript in its entirety.

**Response:** Our ethics statement is available in line 155-157 and line 169 -170 of the revised manscript.

REFERENCES:

*1. Please ensure your references are in the style of PLOS Medicine (ICMJE/Vancouver style). The reference style guide can be found here:*

[*https://eur03.safelinks.protection.outlook.com/?url=http%3A%2F%2Fjournals.plos.org%2Fplosmedicine%2Fs%2Fsubmission-guidelines%23loc-references&amp;data=02%7C01%7Cr.m.douwes%40umcg.nl%7C3c1fd2b5a0244c4aa07008d7ec458e5d%7C335122f9d4f44d67a2fccd6dc20dde70%7C0%7C0%7C637237655802173203&amp;sdata=dh67IOHmNAMveJH2RA%2BvdW8ab5xM8KK2ktZzAI%2F9LE4%3D&amp;reserved=0*](https://eur03.safelinks.protection.outlook.com/?url=http%3A%2F%2Fjournals.plos.org%2Fplosmedicine%2Fs%2Fsubmission-guidelines%23loc-references&amp;data=02%7C01%7Cr.m.douwes%40umcg.nl%7C3c1fd2b5a0244c4aa07008d7ec458e5d%7C335122f9d4f44d67a2fccd6dc20dde70%7C0%7C0%7C637237655802173203&amp;sdata=dh67IOHmNAMveJH2RA%2BvdW8ab5xM8KK2ktZzAI%2F9LE4%3D&amp;reserved=0)*. You can also find a downloadable EndNote file in these guidelines to assist you. Please do a manual check as well, as the EndNote file does not catch everything. Please note that the references will be typeset as they appear so please ensure the format is consistent.*

**Response:** Accordingly, we changed our reference style to the PLOS Medicine style (Vancouver style).

*2. Please put all reference citations in square brackets separated by either commas or dashes with no spaces, e.g. [1,2,3] or [1-3].*

**Response:** Accordingly, we removed all spaces between numbers within the brackets.

*3. Please make sure all references are cited in ascending numerical order in the text.*

**Response:** All references are cited in ascending numerical order int the text.

SUPPORTING INFORMATION:

*1. Please upload your supporting information as individual files, and use the following naming format: S1 Text; S1 Fig; S1 Table, S1 Data, etc. Each file type should start as S1 and increase numerically: S1 Text, S2 Text, etc. Note that all relevant in-text citations and captions will need to be updated accordingly.*

**Response:** Accordingly, we uploaded our supporting information as individual files and updated the in-text citations.

*2. Please ensure that all Supporting Information files use the following format verbatim throughout the manuscript (including legends and all in-text citations): S1 Fig, S1 Table, S1 Text, S1 Data, etc. Each file type should start at 1 and increase numerically. Please note, however, that the file names themselves must use an underscore rather than a space, i.e. S1_Fig.tif, S1_Table.xlsx, S1_Text.doc, S1_Data.xlsx, S1_PRISMA_Checklist.docx, etc.*

**Response:** We changed the file names and in-text citations accordingly.

*3. Please ensure that each supporting information file has a legend at the end of the manuscript file, after the Reference list.*

**Response:** We added a legend for each supporting information file at the end of the manuscript file.

*4. Please note that your submission cannot contain any references to generic "supporting information" files and instead must refer to specific files using our naming convention (S1 Fig, S1 Table, S1 Text, S1 Data, SI PRISMA Checklist, etc.)*

**Response:** We checked our manuscript for correct citation of the supporting information.
